# Challenges in paper-based fluorogenic optical sensing with smartphones

**DOI:** 10.1186/s40580-018-0146-1

**Published:** 2018-05-04

**Authors:** Tiffany-Heather Ulep, Jeong-Yeol Yoon

**Affiliations:** Department of Biomedical Engineering, The University of Arizona, Tucson, AZ 85721 USA

**Keywords:** Paper microfluidics, Point-of-care diagnostics, Fluorescent nanotechnology, Smartphone integration

## Abstract

Application of optically superior, tunable fluorescent nanotechnologies have long been demonstrated throughout many chemical and biological sensing applications. Combined with microfluidics technologies, i.e. on lab-on-a-chip platforms, such fluorescent nanotechnologies have often enabled extreme sensitivity, sometimes down to single molecule level. Within recent years there has been a peak interest in translating fluorescent nanotechnology onto paper-based platforms for chemical and biological sensing, as a simple, low-cost, disposable alternative to conventional silicone-based microfluidic substrates. On the other hand, smartphone integration as an optical detection system as well as user interface and data processing component has been widely attempted, serving as a gateway to on-board quantitative processing, enhanced mobility, and interconnectivity with informational networks. Smartphone sensing can be integrated to these paper-based fluorogenic assays towards demonstrating extreme sensitivity as well as ease-of-use and low-cost. However, with these emerging technologies there are always technical limitations that must be addressed; for example, paper’s autofluorescence that perturbs fluorogenic sensing; smartphone flash’s limitations in fluorescent excitation; smartphone camera’s limitations in detecting narrow-band fluorescent emission, etc. In this review, physical optical setups, digital enhancement algorithms, and various fluorescent measurement techniques are discussed and pinpointed as areas of opportunities to further improve paper-based fluorogenic optical sensing with smartphones.

## Introduction

### Point-of-care diagnostics on silicone-based substrates

Point-of-care (POC) diagnostics are rapid, low-cost, mobile tests that can be conducted in resource-limited environments by little to non-trained personnel. Conducting tests on site allows for faster response time, which in turn increases better opportunities for proper and adequate treatment [1, 2]. The incorporation of microfluidics with POC tests add complexity and versatility to the assays due to controlled flow in discrete spaces, reduction in sample volume, minimized handling of reagents, and ability to run parallel comparison analysis [3].

Silicone-based polymers, specifically polydimethylsiloxane (PDMS), are widely used in the fabrication of microfluidic POC platforms. PDMS provides an optically transparent (230–1100 nm), flexible, nontoxic, and low-cost material. However, when untreated, PDMS surface is a relatively hydrophobic material, which can be problematic in controlling flow especially under low pressure conditions. Also, irreversible protein adsorption to PDMS surface can eventually lead to a nonfunctional device. Although many surface modification techniques have been developed to make PDMS surface hydrophilic, induced hydrophilic states are only temporary: PDMS’ inherent hydrophobicity will return after a period of time [4, 5]. Furthermore, when developing unique infrastructures such as on-chip pumps and valves, PDMS lithography fabrication techniques require a clean room access, which in turn can become complex and expensive.

### POC diagnostics on paper substrates

Alternatively, paper can be used as a microfluidic platform. It is inexpensive, easy to chemically modify (i.e. nitrogen functionalized cellulose is commonly used for biological samples), easy to fabricate, store, and transport [6]. Inkjet processing is also a well-studied and commercially available method for easy functionalization of paper substrates. A widely known and highly used paper-based lateral flow assay (LFA) example is the colorimetric pregnancy test strip. Sandwich immunoassays are the most popularly utilized in paper-based LFAs. Immobilized antibodies on a paper substrate binds with target antigens if present in the specimen. Following, secondary antibodies conjugated to gold nanoparticles act as a reporter and bind to the immobilized antibodies on paper with captured antigens. The resulting positive reaction causes an aggregation-induced pink color appearance [7]. This coloration is due to a spatially dependent optical property of gold nanoparticles, known as the surface plasmon resonance band [8].

However, colorimetric LFAs are binary, i.e. yes-or-no assays, thus difficult to quantify in a reproducible manner. In addition, they show little potential for multiplexing capabilities since they can conduct only one assay at a time. Also, commercially available LFAs require high concentration of target of interest in order to obtain a reliant signal that may not be within the normal or low physiologically relevant levels [7]. As an example, commercially available LFAs for the detection of thyroid stimulating hormone (TSH) has a limit of detection of > 5 mIU/L, which fails to detect the normal and low concentrations of TSH (i.e. hyperthyroidism) in human blood serum [9]. Similarly, commercially available nitrite LFAs for recognizing *Escherichia coli* from urine (for detecting urinary tract infection), as well as *Neisseria gonorrhoeae*, the most common cause of sexually transmitted disease (STD) infection of the urogenital tract, has shown a limit of detection of 10^6^ CFU/mL [10]. Bacteria concentration among the urinary tract infection patients can be as low as 10^2^–10^3^ CFU/mL in adults [11] and even less in children [12]. Also in a thorough analysis of commercial assays for detection of *Cryptosporidium* in fecal samples, the ImmunoCard STAT! LFA platform failed to detect all 12 samples with < 175 organisms per 10 μL sample and had problems with interpretation due to low band intensity [13].

### Fluorescent nanotechnologies lower limit of detection for POC diagnostics

Nanotechnologies combined with fluorescence detection has demonstrated the lowering of limit of detection down to the single cell or picogram protein resolution [14–17]. Implementation of fluorescent nanotechnologies such as quantum dots [18–20] and nanoclusters [21–23] have several advantages over more traditional colorimetric sensors. Nanoscale sensors can easily be tuned to respond to specific excitation wavelengths, by varying shape, size, and length. Nanostructures possess large surface areas for accommodating increased amount of bioreceptor immobilization, and this in turn results in increased sensitivity and much lower limit of detection. Nanoparticles are also highly stable and do not photobleach as easily in comparison to traditional fluorescent dyes [24, 25]. The resulting emission spectrum from these nanoparticles results in increased sensitivity, which in turn shows decreased signal-to-noise ratios. Lastly, materials at which these nanotechnologies are comprised of, such as carbon [26, 27] and gold [28, 29] show superior biocompatibility in complex biological matrices. Fluorogenics in combination with paper-based microfluidic devices, reduces cost, simplifies manufacturability, and improves ease of disposability [30]. Therefore, fluorescent nanotechnologies on paper-based platforms have become an extremely attractive option in biological and chemical sensing [31].

### Smartphone integration into paper-based fluorogenic optical sensing

Evidently, the next step is to integrate these fluorescent nanotechnologies on paper-based platforms in conjunction with smartphone optical sensing, utilizing its flash as a light source, its cameras as an optical detector, and potentially its software application for data processing. As a result, an easy-to-use, point-of-care, yet extremely sensitive handheld platform can be developed. Smartphone integration has shown numerous advantages and opportunities in its use as a detector and user interface platform in POC assays. Smartphones allows for network connection and access, on-board processing, and application in resource-limited settings [30–34].

**Fig. 1:**
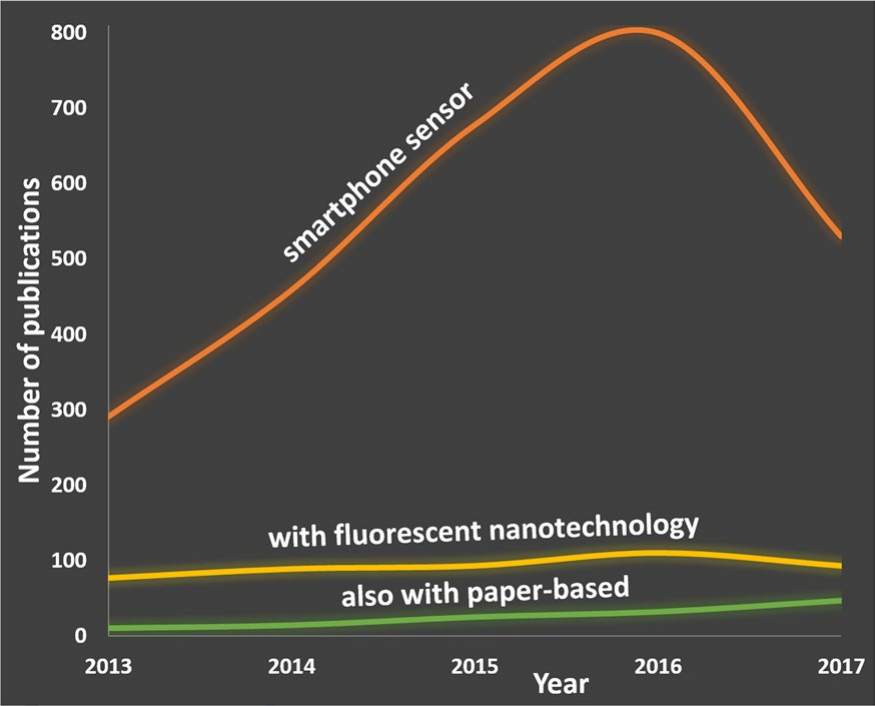
The number of research article publications on smartphone sensor (orange), those with fluorescent nanotechnology (yellow), and above two with paper-based fluorescent (light green) in the past 5 years. Web of science was used

As such, the number of publications in smartphone sensors have significantly increased over the past 5 years, as shown in Fig. 1 (orange line). Expected next steps for smartphone sensing include its demonstration on paper-based platforms, its use in conjunction with fluorescent nanotechnologies, and combination of both, towards improving ease-of-use and sensitivity. However in 2017, the total number of publications in smartphone sensing has started to decrease, for the first time, potentially suggesting challenges in advanced smartphone sensing.

The number of publications demonstrating fluorescent nanotechnology with smartphone sensing also showed a similar trend—slow increase followed by a decrease in 2017 (Fig. 1; yellow line), suggesting complications and challenges in demonstrating fluorescent nanotechnology with smartphone sensing. The number of publications demonstrating all of the above—smartphone sensing of fluorescent nanotechnology on paper-based platform is significantly smaller than overall smartphone sensing (Fig. 1; light green line), again demonstrating its complications, although this number has continued to increase over time.

Although there have been many publications and reviews attesting to these outlined advantages for paper-based fluorescent platforms, there has been a scarce discussion on the challenges, technical limitations, and alternative methods of its fluorescent optical detection, especially in conjunction with smartphone sensing. This paper will review and discuss the problems that are involved when combining fluorescent nanotechnologies, paper-based platforms, and smartphone sensing, as well as the methods that are being developed and practiced to address and overcome them.

## Digital processing

### Digital enhancement

Currently, complementary metal oxide semiconductor (CMOS) array is the most widespread image sensor for smartphones. It utilizes Bayer color filter arrays (CFA) that collect red, green, and blue (RGB) values from a grid-like structure. The raw data pixel values are processed through a demosaicing algorithm, which fill in missing RGB values through interpolation techniques, resulting in an RGB image. Following a demosaic algorithm, a denoising algorithm is then applied [35, 36]. Data image processing widely varies between smartphone models and brands. Along with data processing, physical properties between CMOS image sensors also vary by models and brands. Recently, Fontaine [37] released a well-organized and detailed publication outlining the different CMOS schematics among smartphone models and brands as well as the evolution of the technology over the years. Such variances include the spatial resolution between metal aperture walls, color filters used, and optical stacking thickness.

In most paper-based point-of-care assays that utilize fluorescence, pixel intensities are extracted and converted into a concentration of a target of interest, i.e., molecule [38–40], protein [23, 41], whole organism [42], or nucleic acid [43, 44]. When trying to control and maintain constant lighting, on-board default camera settings on smartphones can be problematic, as they are constantly trying to adjust white balance, focus and exposure during multiple assays. An especially concerning problem is the camera’s ability to resolve very small points of interests such as the test line on a LFA or fluorophore-loaded areas on paper microfluidic devices [30].

To further refine fluorescent images captured, it is also common to apply digital filters. With use of digital filters there is no physical adjustments required to the overall optical setup. This in turn offers a low cost and simple method for correcting undesirable flaws. In fluorescence imaging, excessive crosstalk, which is the inconsistent recognition from left to right views resulting in a blurry effect, is a typical correction to address [45, 46].

There has also been numerous publications investigating better extraction and interpretation of measured RGB pixel values from the smartphone captured images on paper-based platforms. Different color spaces have been deployed to enhance paper-based pixel intensities [34]. In Shen et al.'s color conversion analysis and quantification of colorimetric pH test strips [47], a more sensitive and accurate method was developed utilizing a 12 region reference chart to account for variability in lighting conditions. The group also alluded to its use in fluorescence paper microfluidic data, although it has not yet been demonstrated. Yetisen et al.'s image processing algorithm of transforming RGB values into non-linear, linear, tristimulus, then into 2D CIE 1921 chromaticity space [31] showed improvements in mitigating variability due to focus, angle, lighting, and sensor type. The applied algorithm was also demonstrated using two different model phones, iPhone 5 (8 MP camera) and Samsung I5500 Galaxy 5 (2 MP camera), therefore proving interphone adaptability. McCracken et al. [48] demonstrated the use of a triple-reference point normalization as well as fast-Fourier transform pre-processing using two different smartphone models (iPhone 5S and Nexus 5X). The developed image process reduced spatial variability due to inconsistent paper surface, shadows, and uneven background reflectance for paper-based microfluidic assays using absorbance, quenching, and scattering measurements.

### Ratiometric FRET

Fluorescence resonance energy transfer (FRET) is the mechanism in which a fluorescent signal is produced due to a transfer of electrons from a donor fluorophore to an acceptor fluorophore that is within Angstrom proximal distances. As a result, two distinctive wavelength peaks are generated and can be measured in a ratiometric manner. In order for this to be efficiently monitored, peak excitation and emission wavelengths must be sufficiently separated, while having an overlap in donor emission and acceptor excitation spectra [49–51].

Ratiometric measurement is an attractive property to measure due to its inherent ability to correct for environmental factors (such as varied lighting conditions and/or optical transparency of medium; especially useful on paper-based platforms) and to self-calibrate [52, 53]. Fluorescent dyes, although have been popularly used to demonstrate FRET-based ratiometric assays, can be easily susceptible to photobleaching [54]. More recently, the use of quantum dots [53, 55, 56] and gold nanoclusters [21] have been more favorable choices for FRET-based sensors due to its photostability and superior intensity from background and undesired autofluorescence [57].

**Fig. 2:**
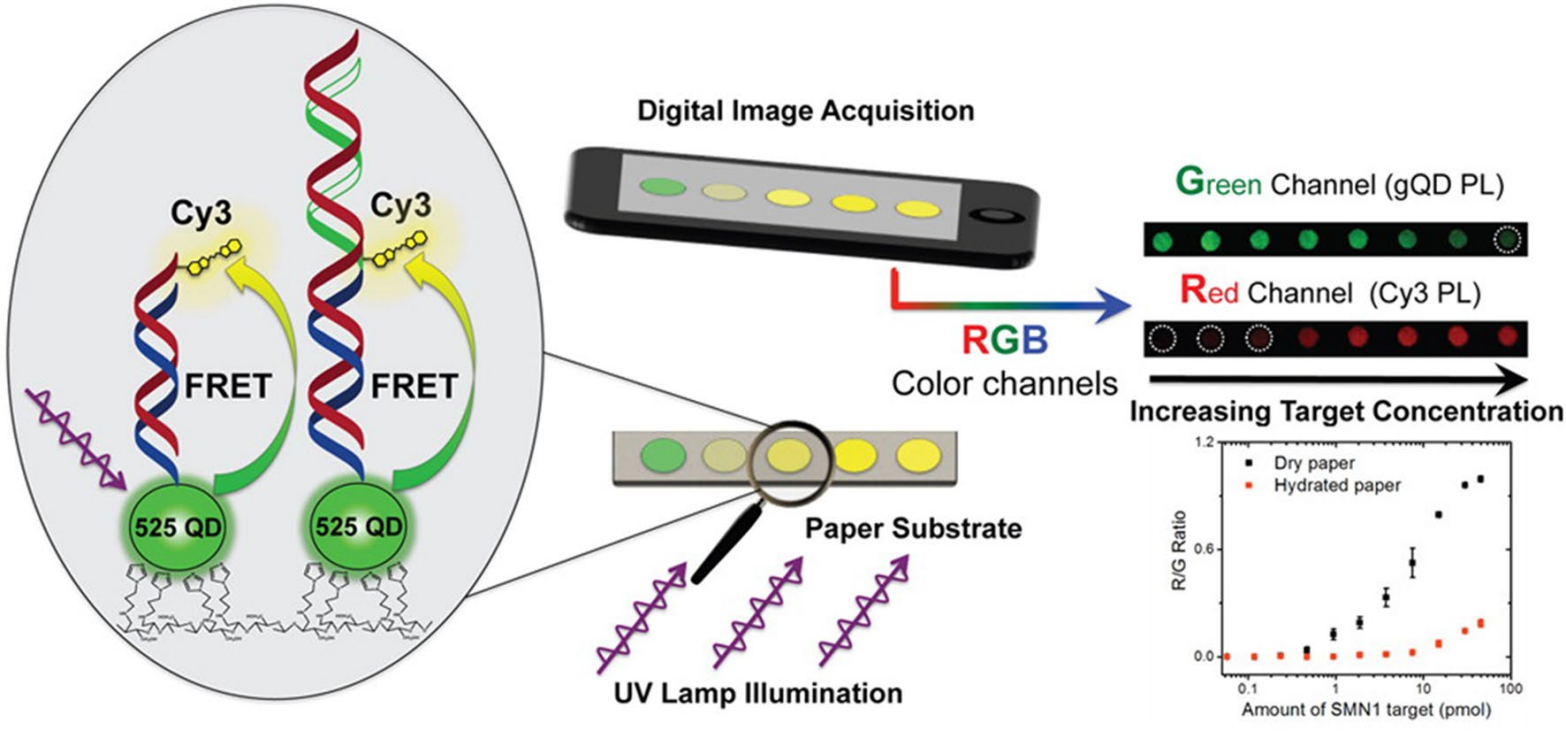
Mechanism and process of ratiometric FRET-sensitized emission to detect nucleic acid hybridization (reproduced from [52] with permission, © 2014 American Chemical Society)

**Fig. 3:**
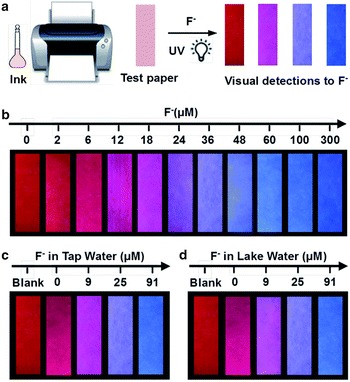
Ratiometric FRET to detect fluoride concentration of water samples on paper-based substrates: **a** test paper preparation; **b** F^-^ detection in DI water; **c** in tap water; **d** in lake water (reproduced from [58], published by Royal Society of Chemistry with open access)

With regards to its applications in smartphone-based paper platforms, ratiometric fluorescent intensities can be easily monitored by simple splitting of red, green, and blue channels in a captured image. Wang et al. [40] measured 803 nm fluorescent intensity in relation to blue emission of upconversion nanoparticles (NaYF_4_:Yb and Tm@NaYF_4_) on paper to detect organophosphate nerve agents. As depicted in Fig. 2, Noor and Krull [52] demonstrated the use of a smartphone where associated green and red pixel values were measured to monitor a nucleic acid hybridization assay. An inversely related relationship was shown with correlated FRET-based transduction of donor green-emitting quantum dots and acceptor Cy3 fluorescent dye acceptor through a R/G (red over green) ratio. Yu et al. [58] prepared a ratiometric fluorescent test paper for visualization and quantification of fluoride ions in environmental waters with the use of CdTe quantum dots. As shown in Fig. 3 red and blue fluorescence intensities were inversely related with the addition of fluoride ions.

## Hardware

### Light sources

Paper-based microfluidics has proven the ability to provide flow without the use of external pumps or high voltage–power (necessary in most silicone or PDMS-based microfluidic devices), but rather by spontaneous capillary action amongst paper fibers (also known as wicking). With the addition of fluorescent detection method for an analyte (protein, cell, or nucleic acid), a light source is required. When using fluorogenics, specific excitation wavelengths are important in order to obtain the desired emission spectra. This being the case, most smartphone-based fluorescent assays incorporate external light sources, the most popular being a handheld UV lamp or separate LED [52, 59, 60]. Taking this into account, various platforms have been designed to accessorize and power an external light source with a specific wavelength needed for fluorescent sensing on paper platforms.

3D-printed plastic attachments that are custom-fitted to a smartphone are widely used, where a built-in LED can be housed along with additional reflectors, collimators, and filters to improve signals [61–63]. Such attached enclosure provides a controlled environment in terms of lighting and spatial distances to improve reproducibility between assays. However, smartphone dimensionality and availability vary greatly between manufacturers and models (also by the use of protective cases and covers), making custom attachments undesirable due to its poor adaptability. Along with using an external light source, an external power source is also required.

**Fig. 4:**
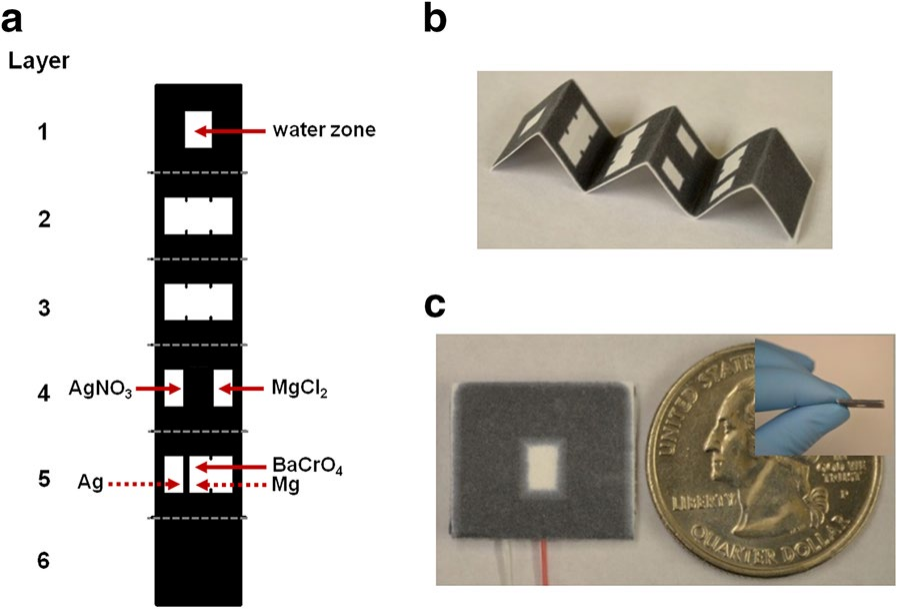
Self-powered paper microfluidic device, utilizing origami paper and galvanic cell, for enzymatic (alkaline phosphatase) fluorescent assay with smartphone detection: **a** device layout; **b** paper folding; **c** top and side view of final assembly (reprinted from [64] with permission, © 2014 AIP Publishing)

An innovative method of powering an external LED with the required excitation wavelength is the integration of a galvanic cell, also known as a fluidic battery. Fluidic batteries are foldable and stackable hydrophilic paper layers with printed hydrophobic wax barriers as shown in Fig. 4. With the application of a water droplet, the fluidic battery powers an LED until it is run dried. The main requirements of a fluidic battery are (1) electrolytes (i.e. AgNO_3_, AgCl_3_, AgNO_2_, or MgCl_2_), (2) electrodes (i.e. silver metals, aluminum metals, or magnesium foil), (3) salt bridges (i.e. containing NaNO_3_), and (4) conductive connections (i.e. copper tape) [64–66].

Instead of using an external light source, the white on-board LED flash on a smartphone can be also used as an excitation source to create a fully smartphone-integrated platform [67–70]. However, band-pass or low-pass fitted filters are often used to separate out exclusive wavelengths for excitation, as smartphone flashes generate “white” light [69, 71].

### Optical filters

Typical means of isolating fluorescence emission include the use of optical filters. Using a low-, high-, band-pass, or other filters inserted prior to the receiving detector (i.e. CMOS array sensor, the most common camera used for smartphones) increases selectivity of emitted fluorescent light. Not only can these filters differentiate wavelengths, but can also serve as a mechanical method for controlling unwanted scattering and diffracting light. Two types of optical filters are commonly used for fluorescence detection. The first type is an absorption filter, in which absorption at the excitation wavelength is desirable and absorption at the emission wavelength is undesirable. In contrast, an interference filter has low absorption at the excitation wavelength and high absorption at the emission wavelength.

**Fig. 5:**
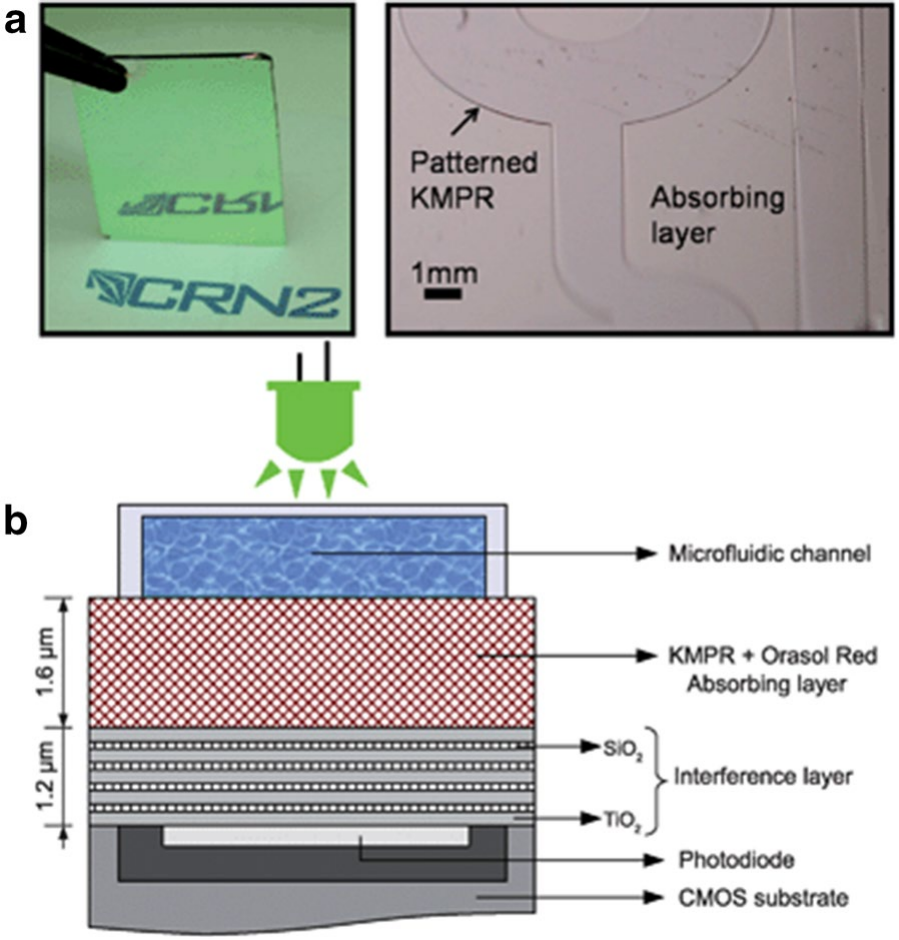
Interference filter added on a microfluidic device: **a** interference filter deposited on glass substrate (left) and patterned microfluidic channels on absorbing layer (right); **b** overall schematics (reproduced from [73] with permission, © 2009 Royal Society of Chemistry)

Interference filters are comprised of multiple thin layers of dielectric material with different refractive indices. Selectivity of wavelength of interest is dictated by the mechanistic pathways that light travels at the fabricated boundary layers. UV excitation is a very common wavelength regime that fluorescence nanotechnology utilizes (most notably quantum dots). This can be very problematic since cellulose paper’s autofluorescence is optimally excited with UV. Therefore, numerous UV filters have been developed to optimize the use of UV excitation. Other filters include a filter developed by Dattner and Yadid-Pecht [72], which is a transparent, poly-acrylic acid (PAA) emission filter, mounted on a low-light CMOS array sensor for selecting red fluorescence. Similarly, Richard et al. [73] fabricated a nine layer interference filter to select 650 nm red emission from quantum dots with 532 nm excitation wavelength. The final filter was integrated into a silicone-based (thus optically transparent) microfluidic device equipped with a CMOS array sensor (Fig. 5).

In comparison, absorption filters are comprised of one single layer. Absorptivity can be adjusted by the overall thickness of the filter and can be modeled using the Beer–Lambert law, *A* = *ε* × *l* × *c*, where *ε* is the characteristic molar absorptivity of the filter, *l* is the path length or thickness of the filter, and *c* is the concentration of the absorbing material [72, 74]. Absorption properties can also be controlled by the addition of dyes such as Sudan II [75, 76], Orasol Red BL, KMPR^®^ 1005 epoxy-based photoresist [73], Aptina green1, and Aptina red1 [77].

Other unique filters can also be used to further enhance fluorescent images collected. Lee et al. [78] demonstrated the use of a silo-filter comprised of metal lattices, which were used as dividends for individual pixels and light guides for fluorescent light to penetrate an absorptive, thick filter layer. The silo-filter’s metal surfaces contributes an enhanced scattering and reflectance effect, improving transmittance and overall background rejection. Photonic structures is also another widely used filtering component for controlling fluorescence emission by means of specifically patterned surfaces on gold [79] and plastic [80]. In an optofluidic chip developed by Ricciardi et al. [81], a fluorescence immunoassay was demonstrated for the detection of actin-actin antibody complexes with superior repeatability and limit of detection. They utilized unique photonic structures for controlled light radiation into a fluorescence microscope apparatus. Similarly, Schudel et al. [82] developed a silicone-based microfluidic chip array that utilized actuate-to-open valve mixing and photonic crystal nanostructures to detect the binding of IgG to various proteins in an immunofluorescent assay using a charge-coupled device (CCD) array sensor.

## Addressing autofluorescence

It is notoriously known that cellulose-based paper substrates exhibit autofluorescence. Cellulose paper is strongly excited with UV, followed by blue, generating blue to green emissions [83]. Therefore an unwanted background autofluorescence, along with paper’s reflection (back scattering), must be addressed. Also with the use of biological samples, autofluorescence and back scattering light from paper surfaces can be even more problematic [84, 85].

### Pulse excitation and time-resolved detection

**Fig. 6:**
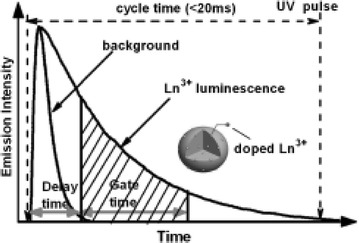
Mechanism of time-resolved fluorescence as a means of eliminating short lifetime background and autofluorescence (reproduced from [86] with permission, (C) 2011 John Wiley & Sons)

Traditional photo-detection instrumentations are designed to receive photons continuously during the excitation period. As a result, mitigation of unwanted background noise (especially autofluorescence) can be difficult. Pulse excitation and time-resolved detection are methods in which fluorophores are acutely exposed to an excitation light. From the collected fluorescent decay, a lifetime value can be determined that is unique to a fluorophore of interest. The measured lifetime can be crucial in resolving between background autofluorescence and detection-related fluorescence. Therefore, designation and separation of timed windows, short lifetime decays of autofluorescence (delay time) and long lifetime decays of fluorophores of interest (gate time), can be easily distinguished and collected as seen in Fig. 6 [54, 84, 86].

**Fig. 7:**
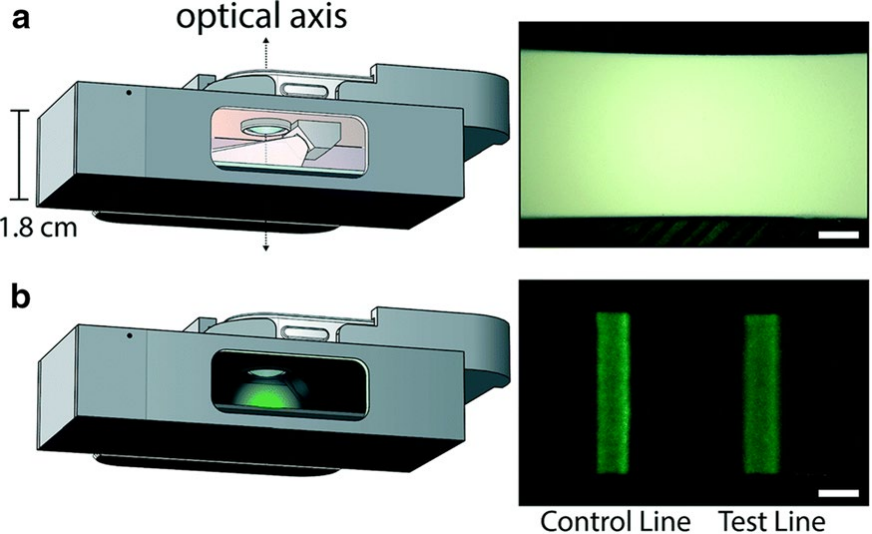
Smartphone attachment utilizing the onboard “white” LED flash as a light source, using pulse excitation to address the paper's autofluorescence, for detecting human chorionic gonadotropin (hCG) on LFA strip: **a** smartphone's flash is turned on for short excitation; **b** flash is turned off showing luminescence imaging (reproduced from [88] with permission, © 2017 Royal Society of Chemistry)

Ju et al. [86] developed lanthanide (Ln^3+^) doped GdF_3_ nanocrystals that successfully detected avidin protein under time-resolved fluorometric measurements with promising applications in immunoassays, multiplexing, and DNA hybridization. Wang et al. [54] demonstrated the use of time-resolved fluorescence in conjunction with ratiometric measurements using a smartphone on a paper-based platform to detect dipicolinic acid (DPA), a biomarker for anthrax presence. Lanthanide-terbium (Ln-Tb) and -europium (Ln-Eu) doped fluorescent crystals (Tb/DPA@SiO_2_-Eu/GMP) when exposed to a 254 nm UV lamp and DPA, resulted in an indicating red fluorescence, as opposed to a control green fluorescence signal. The spectra was further refined by using a delay time of 50 μs and gate time of 2 ms to avoid autofluorescence. Similarly, Kim et al. [87] demonstrated the use of time-resolved fluorescence measurements to study the enhanced FRET efficiency and increased fluorescent lifetime of immobilized quantum dots on a paper platform in comparison to a solution assay. The four-fold enhancement in FRET rate was concluded and attributed to the decreased average distance between quantum dot donor and acceptor dye for the paper-based platform. Overall, colored digital images were captured and analyzed under a 405 nm LED to analyze trypsin proteolytic activity and inactivity in the presence of aprotinin inhibition. Paterson et al. [88] utilized a smartphone time-gated imaging application to capture images at set intervals after pulse excitation to detect human chorionic gonadotropin (hCG) with strontium aluminate nanoparticles on a LFA (Fig. 7).

### Autofluorescence indexing

Up until now, what has been discussed were methods practiced to avoid, normalize, or subtract the inherent autofluorescence of paper matrices. In a recent publication done by Shah and Yager [89], a systematic “autofluorescence index” was proposed using excitation–emission matrices for screening and selecting paper substrates for low autofluorescence when developing assays. Conventionally, primarily and solely spectral overlap is considered between target-induced fluorescence and paper over fluorescence. In Shah and Yager study, 12 different paper matrices, including nitrocellulose, glass fiber, and cellulose that are commonly used, were measured and evaluated using developed autofluorescence index equations. The proposed quantification of autofluorescence was further investigated and demonstrated using a quantum dot lateral flow immunoassay for detection of influenza A nucleoprotein. It was concluded that paper matrices with lower calculated autofluorescence indices had lower limits of detections.

### Use of NIR

Autofluorescence of paper can also be avoided by using longer wavelengths, e.g. near infrared (NIR) or infrared (IR) [90–93]. Yu and White [94] observed that background autofluorescence of paper was reduced using 785 nm excitation in assaying Rhodamine 6G, organophosphate malathion, heroin, and cocaine from a surface-enhanced Raman spectroscopy dipstick swab. Similarly, Ju et al. [95] found using longer IR or NIR wavelengths as the excitation source reduced autofluorescence as well as undesired back scattering. Their paper-based platform utilizing lanthanide-doped LiYF_4_ upconversion nanoparticles demonstrated a limit of detection of 3.6 fmol of DNA. Doughan et al. [96] used a 980 nm NIR excitation to reduce background noise that usually corresponds with UV or visible wavelength excitation.

## FUTURE OUTLOOK: Contact CMOS fluorescence imager

**Fig. 8:**
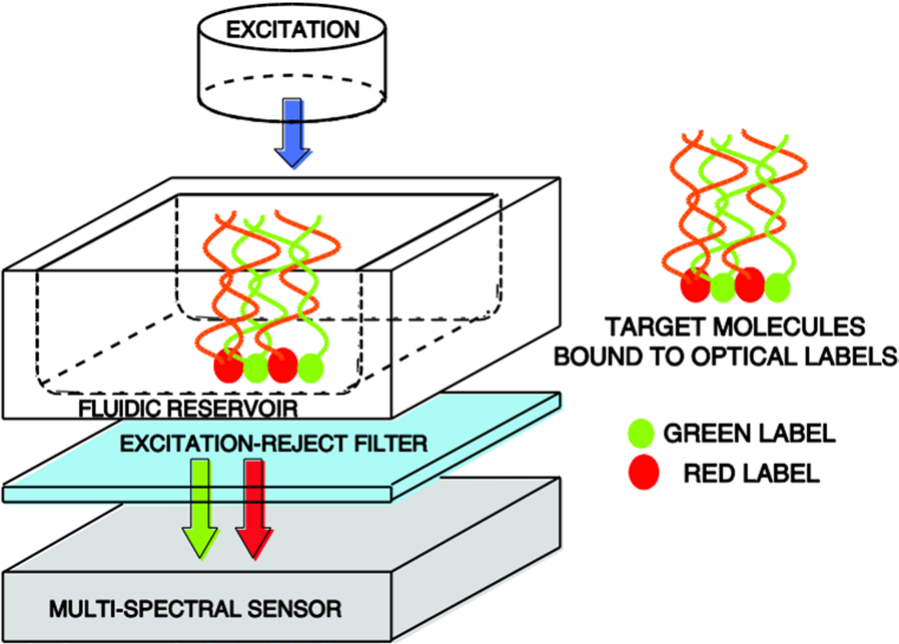
Contact CMOS fluorescent imaging system set up (reproduced from [84], published by MDPI in open access)

In more recent years, topics revolving fluorescence-based detection discuss the options of totally bypassing the use of a smartphone detectors and leaning towards more conventional optical equipment. This can be done by using a contact CMOS fluorescence imager integrated into biosensors [97–100]. With manufacturing costs and overall prices of CMOS imaging sensors low enough, the manufactured parts have become recyclable or disposable components. Therefore, the timeliness of integrating it as a detector in point-of-care assays is ideal. A contact CMOS fluorescence imager is made of a CMOS image sensor chip, a thin-film absorption filter, and a fiber optic plate. When mounted underneath an ultra-thin transparent material such as a glass or PDMS chip (Fig. 8), weak fluorescence signals can be efficiently collected [84]. However, its demonstration on paper-based platform is yet to be seen.

**Fig. 9:**
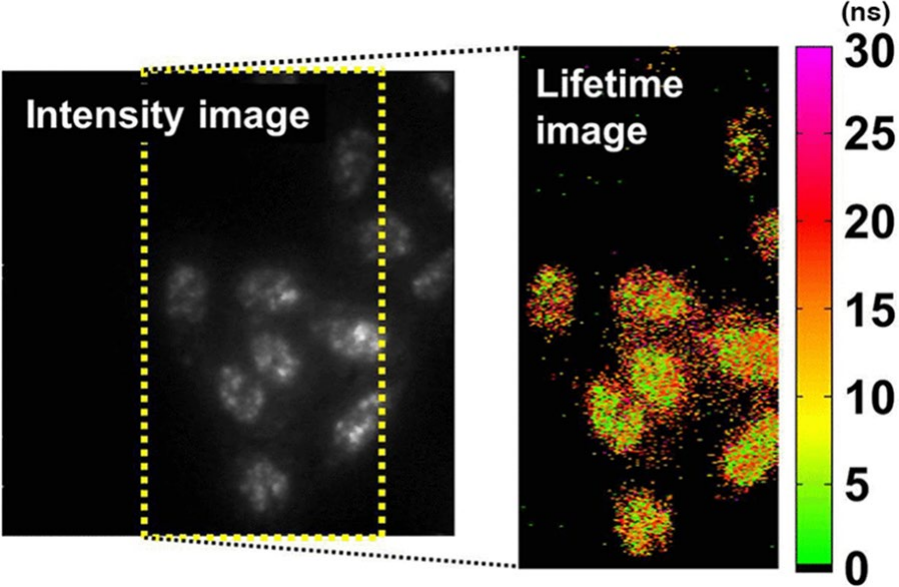
Lifetime color mapping of fluorescent-stained cells using contact CMOS sensor (reproduced from [101] with permission, © 2016 IEEE)

Such systems have been demonstrated to resolve highly comparable fluorescent images of live cells [102–104] (Fig. 9). Salama et al. [45] compared commercially available digital fluorescence readers to CMOS-based technologies and determine a three times lower detection limit in a pyrosequencing DNA assay. Zheng et al. [105] demonstrated a 660 nm resolution ePetri dish prototype constructed out of Lego blocks (holder), a smartphone (not as a detector but as a light source) and a CMOS imager. More recently, Seo et al. [101] developed a CMOS-based sensor that resulted in fluorescent lifetime images of cells labeled with DAPI fluorescent dye and quantum dots. Jain et al. [106] evaluated the use of a digital CMOS imaging system in a quantum dot immunofluorescent assay capable of multiplexing. The portable, commercially available ArrayCAM™ was used to demonstrate the quantification of three different fluorophores labeled for IgG, IgA, and IgM due to the discrete emission quantum dot spectra.

However, its implementation in paper-based assays is uncertain due to the spatial limitation between sample and CMOS sensor—how can we maintain precise distance between paper and CMOS sensor? In addition, the platform must be transparent or have efficient transmittance, while paper is obviously not. Overall, the CMOS imager technology shows promising wide field of view, lens-free translation for automation, and downsizing of traditional fluorescent optical setups. In addition, automatic correction, focus, and white balance variability that is normally associated with smartphones can be finely controlled or completely bypassed with the use of contact CMOS technology [79, 84]. However, such demonstration on paper platforms is yet to be seen, leaving smartphone sensing as a better choice for the time being.

## Conclusion

Overall, fluorogenic-based detection, especially in conjunction with nanotechnology, has shown increasing trends in translation to low-cost and easy-to-use paper-based platforms due to its superior emission intensities, assay specificity, time-dependent resolvement, and superior storage life. Smartphone-based optical sensing of fluorescence emission is of recent development and has shown numerous technical and physical limitations, especially on paper platforms. However, recent implementation of filters, advanced image processing, and unique platform development show improved results to better address such challenges. Hopefully in the near future, the benefits of contact CMOS imagers could also be incorporated into smartphone-based fluorescence sensing on paper-based platforms.

## References

[1] Posthuma-Trumpie GA, Korf J, van Amerongen A (2009). Lateral flow (immuno)assay: its strengths, weaknesses, opportunities and threats. A literature survey. Anal. Bioanal. Chem..

[2] Yager P, Edwards T, Fu E, Helton K, Nelson K, Tam MR, Weigl BH (2006). Microfluidic diagnostic technologies for global public health. Nature.

[3] Sia SK, Kricka LJ (2008). Microfluidics and point-of-care testing. Lab Chip.

[4] Kumar S, Kumar S, Ali MA, Anand P, Agrawal VV, John R, Maji S, Malhotra BD (2013). Microfluidic-integrated biosensors: prospects for point-of-care diagnostics. Biotechnol. J..

[5] Zhou J, Khodakov DA, Ellis AV, Voelcker NH (2011). Surface modification for PDMS-based microfluidic devices. Electrophoresis.

[6] Scida K, Li B, Ellington AD, Crooks RM (2013). DNA detection using origami paper analytical devices. Anal. Chem..

[7] Zhao W, van den Berg A (2008). Lab on paper. Lab Chip.

[8] Yoon JY (2016). Introduction to biosensors: from electric circuits to immunosensors.

[9] You DJ, Park TS, Yoon JY (2013). Cell-phone-based measurement of TSH using Mie scatter optimized lateral flow assays. Biosens. Bioelectron..

[10] Cho S, Park TS, Nahapetian TG, Yoon JY (2015). Smartphone-based, sensitive µPAD detection of urinary tract infection and gonorrhea. Biosens. Bioelectron..

[11] Wilson ML, Gaido L (2004). Laboratory diagnosis of urinary tract infections in adult patients. Clin. Infect. Dis..

[12] Jodal U, Lindberg U, Lincoln K (2008). Level diagnosis of symptomatic urinary tract infections in childhood. Acta Paediatr..

[13] Johnston SP, Ballard MM, Beach MJ, Causer L, Wilkins PP (2003). Evaluation of three commercial assays for detection of Giardia and Cryptosporidium organisms in fecal specimens. J. Clin. Microbiol..

[14] Kinkhabwala A, Yu Z, Fan S, Avlasevich Y, Müllen K, Moerner WE (2009). Large single-molecule fluorescence enhancements produced by a bowtie nanoantenna. Nat. Photonics.

[15] Jin H, Heller DA, Kalbacova M, Kim JH, Zhang J, Boghossian AA, Maheshri N, Strano MS (2010). Detection of single-molecule H_2_O_2_ signalling from epidermal growth factor receptor using fluorescent single-walled carbon nanotubes. Nat. Nanotechnol..

[16] McGuinness LP, Yan Y, Stacey A, Simpson DA, Hall LT, Maclaurin D, Prawer S, Mulvaney P, Wrachtrup J, Caruso F, Scholten RE, Hollenberg LCL (2011). Quantum measurement and orientation tracking of fluorescent nanodiamonds inside living cells. Nat. Nanotechnol..

[17] Tabassum S, Al-Asbahy WM, Afzal M, Arjmand F, Khan RH (2012). Interaction and photo-induced cleavage studies of a copper based chemotherapeutic drug with human serum albumin: spectroscopic and molecular docking study. Mol. BioSyst..

[18] Li Z, Wang Y, Wang J, Tang Z, Pounds JG, Lin Y (2010). Rapid and sensitive detection of protein biomarker using a portable fluorescence biosensor based on quantum dots and a lateral flow test strip. Anal. Chem..

[19] Hansen JA, Wang J, Kawde AN, Xiang Y, Gothelf KV, Collins G (2006). Quantum-dot/aptamer-based ultrasensitive multi-analyte electrochemical biosensor. J. Am. Chem. Soc..

[20] Zajac A, Song D, Qian W, Zhukov T (2007). Protein microarrays and quantum dot probes for early cancer detection. Colloids Surf. B.

[21] Zhuang M, Ding C, Zhu A, Tian Y (2014). Ratiometric fluorescence probe for monitoring hydroxyl radical in live cells based on gold nanoclusters. Anal. Chem..

[22] Wang Y, Ge L, Wang P, Yan M, Ge S, Li N, Yu J, Huang J (2013). Photoelectrochemical lab-on-paper device equipped with a porous Au-paper electrode and fluidic delay-switch for sensitive detection of DNA hybridization. Lab Chip.

[23] Anjana RR, Devi JSA, Jayasree M, Aparna RS, Aswathy B, Praveen GL, Lekha GM, Sony G (2018). S, N-doped carbon dots as a fluorescent probe for bilirubin. Microchim. Acta.

[24] Ferrari M (2005). Cancer nanotechnology: opportunities and challenges. Nat. Rev. Cancer.

[25] Wu M, Lai Q, Ju Q, Li L, Yu HD, Huang W (2018). Paper-based fluorogenic devices for in vitro diagnostics. Biosens. Bioelectron..

[26] Fiorito S, Serafino A, Andreola F, Togna A, Togna G (2006). Toxicity and biocompatibility of carbon nanoparticles. J. Nanosci. Nanotechnol..

[27] Murugesan S, Park TJ, Yang H, Mousa S, Linhardt RJ (2006). Blood compatible carbon nanotubes—nano-based neoproteoglycans. Langmuir.

[28] Shukla R, Bansal V, Chaudhary M, Basu A, Bhonde RR, Sastry M (2005). Biocompatibility of gold nanoparticles and their endocytotic fate inside the cellular compartment: a microscopic overview. Langmuir.

[29] Hu X, Gao X (2011). Multilayer coating of gold nanorods for combined stability and biocompatibility. Phys. Chem. Chem. Phys..

[30] Martinez AW, Phillips ST, Carrilho E, Thomas SW, Sindi H, Whitesides GM (2008). Simple telemedicine for developing regions: camera phones and paper-based microfluidic devices for real-time, off-site diagnosis. Anal. Chem..

[31] Yetisen AK, Martinez-Hurtado JL, Garcia-Melendrez A, da Cruz Vasconcellos F, Lowe CR (2014). A smartphone algorithm with inter-phone repeatability for the analysis of colorimetric tests. Sens. Actuators B-Chem..

[32] Xu X, Akay A, Wei H, Wang S, Pingguan-Murphy B, Erlandsson BE, Li X, Lee W, Hu J, Wang L, Xu F (2015). Advances in smartphone-based point-of-care diagnostics. Proc. IEEE.

[33] McCracken KE, Yoon JY (2016). Recent approaches for optical smartphone sensing in resource-limited settings: a brief review. Anal. Meth..

[34] Vashist SK, Mudanyali O, Schneider EM, Zengerle R, Ozcan A (2014). Cellphone-based devices for bioanalytical sciences. Anal. Bioanal. Chem..

[35] Paliy D, Foi A, Bilcu R, Katkovnik V (2008). Denoising and interpolation of noisy Bayer data with adaptive cross-color filters. Proc. SPIE.

[36] Jin X, Liu Z, Chen J (2010). CMOS vision sensor with fully digital image process integrated into low power 1/8-inch chip. Chin. Opt. Lett..

[37] R. Fontaine, A survey of enabling technologies in successful consumer digital imaging products. In: Proceedings of the international image sensors workshop, Hiroshima, Japan, 30 May—2 June 2017 (2017)

[38] Qin SJ, Yan B (2018). The point-of-care colorimetric detection of the biomarker of phenylamine in the human urine based on Tb^3+^ functionalized metal-organic framework. Anal. Chim. Acta.

[39] Xu H, Zhang K, Liu Q, Liu Y, Xie M (2017). Visual and fluorescent detection of mercury ions by using a dually emissive ratiometric nanohybrid containing carbon dots and CdTe quantum dots. Microchim. Acta.

[40] Wang X, Wang S, Huang K, Liu Z, Gao Y, Zeng W (2017). A ratiometric upconversion nanosensor for visualized point-of-care assay of organophosphonate nerve agent. Sens. Actuators B-Chem..

[41] Das P, Krull UJ (2017). Detection of a cancer biomarker protein on modified cellulose paper by fluorescence using aptamer-linked quantum dots. Analyst.

[42] Weng X, Neethirajan S (2017). Aptamer-based fluorometric determination of norovirus using a paper-based microfluidic device. Microchim. Acta.

[43] Li B, Zhou X, Liu H, Deng H, Huang R, Xing D (2018). Simultaneous detection of antibiotic resistance genes on paper-based chip using [Ru(phen)2d ppz]2+ turn-on fluorescence probe. ACS Appl. Mater. Interfaces..

[44] Seok Y, Joung HA, Byun JY, Jeon HS, Shin SJ, Kim S, Shin YB, Han HS, Kim MG (2017). A paper-based device for performing loop-mediated isothermal amplification with real-time simultaneous detection of multiple DNA targets. Theranostics.

[45] Salama K, Eltoukhy H, Hassibi A, Gamal AE (2004). Modeling and simulation of luminescence detection platforms. Biosens. Bioelectron..

[46] Li XF, Wang QH, Li DH, Wang AH (2011). Image processing to eliminate crosstalk between neighboring view images in three-dimensional lenticular display. J. Disp. Technol..

[47] Shen L, Hagen JA, Papautsky I (2012). Point-of-care colorimetric detection with a smartphone. Lab Chip.

[48] McCracken KE, Angus SV, Reynolds KA, Yoon JY (2016). Multimodal imaging and lighting bias correction for improved μPAD-based water quality monitoring via smartphones. Sci. Rep..

[49] Sekar RB, Periasamy A (2003). Fluorescence resonance energy transfer (FRET) microscopy imaging of live cell protein localizations. J. Cell Biol..

[50] Pollok BA, Heim R (1999). Using GFP in FRET-based applications. Trends Cell Biol..

[51] Jares-Erijman EA, Jovin TM (2003). FRET imaging. Nat. Biotechnol..

[52] Noor MO, Krull UJ (2014). Camera-based ratiometric fluorescence transduction of nucleic acid hybridization with reagentless signal amplification on a paper-based platform using immobilized quantum dots as donors. Anal. Chem..

[53] Díaz SA, Giordano L, Jovin TM, Jares-Erijman EA (2012). Modulation of a photoswitchable dual-color quantum dot containing a photochromic FRET acceptor and an internal standard. Nano Lett..

[54] Wang QX, Xue SF, Chen ZH, Ma SH, Zhang S, Shi G, Zhang M (2017). Dual lanthanide-doped complexes: the development of a time-resolved ratiometric fluorescent probe for anthrax biomarker and a paper-based visual sensor. Biosens. Bioelectron..

[55] Tyrakowski CM, Snee PT (2014). Ratiometric CdSe/ZnS quantum dot protein sensor. Anal. Chem..

[56] Wang K, Qian J, Jiang D, Yang Z, Du X, Wang K (2015). Onsite naked eye determination of cysteine and homocysteine using quencher displacement-induced fluorescence recovery of the dual-emission hybrid probes with desired intensity ratio. Biosens. Bioelectron..

[57] Algar WR, Massey M, Krull UJ (2009). The application of quantum dots, gold nanoparticles and molecular switches to optical nucleic-acid diagnostics. Trends Anal. Chem..

[58] Yu X, Yang L, Zhao T, Zhang R, Yang L, Jiang C, Zhao J, Liu B, Zhang Z (2017). Multicolorful ratiometric-fluorescent test paper for determination of fluoride ions in environmental water. RSC Adv..

[59] Dou M, Dominguez DC, Li X, Sanchez J, Scott G (2014). A versatile PDMS/paper hybrid microfluidic platform for sensitive infectious disease diagnosis. Anal. Chem..

[60] Caglayan MG, Sheykhi S, Mosca L, Anzenbacher P (2016). Fluorescent zinc and copper complexes for detection of adrafinil in paper-based microfluidic devices. Chem. Commun..

[61] Yeo SJ, Choi K, Cuc BT, Hong NN, Bao DT, Ngoc NM, Le MQ, Hang NLK, Thach NC, Mallik SK, Kim HS, Chong CK, Choi HS, Sung HW, Yu K, Park H (2016). Smartphone-based fluorescent diagnostic system for highly pathogenic H5N1 viruses. Theranostics.

[62] Koydemir HC, Gorocs Z, Tseng D, Cortazar B, Feng S, Chan RYL, Burbano J, McLeod E, Ozcan A (2015). Rapid imaging, detection and quantification of Giardia lamblia cysts using mobile-phone based fluorescent microscopy and machine learning. Lab Chip.

[63] Hossain A, Canning J, Ast S, Rutledge PJ, Yen TL, Jamalipour A (2015). Lab-in-a-phone: smartphone-based portable fluorometer for pH measurements of environmental water. IEEE Sens. J..

[64] Koo Y, Sankar J, Yun Y (2014). High performance magnesium anode in paper-based microfluidic battery, powering on-chip fluorescence assay. Biomicrofluidics.

[65] Thom NK, Yeung K, Pillion MB, Phillips ST (2012). “Fluidic batteries” as low-cost sources of power in paper-based microfluidic devices. Lab Chip.

[66] Thom NK, Lewis GG, Yeung K, Phillips ST (2014). Quantitative fluorescence assays using a self-powered paper-based microfluidic device and a camera-equipped cellular phone. RSC Adv..

[67] McCracken KE, Tat T, Paz V, Yoon JY (2017). Smartphone-based fluorescence detection of bisphenol A from water samples. RSC Adv..

[68] Long KD, Woodburn EV, Le HM, Shah UK, Lumetta SS, Cunningham BT (2017). Multimode smartphone biosensing: the transmission, reflection, and intensity spectral (TRI)-analyzer. Lab Chip.

[69] Petryayeva E, Algar WR (2016). A job for quantum dots: use of a smartphone and 3D-printed accessory for all-in-one excitation and imaging of photoluminescence. Anal. Bioanal. Chem..

[70] Hossain MA, Canning J, Ast S, Cook K, Rutledge PJ, Jamalipour A (2015). Combined “dual” absorption and fluorescence smartphone spectrometers. Opt. Lett..

[71] Canning J, Lau A, Naqshbandi M, Petermann I, Crossley MJ (2011). Measurement of fluorescence in a rhodamine-123 doped self-assembled “giant” mesostructured silica sphere using a smartphone as optical hardware. Sensors.

[72] Dattner Y, Yadid-Pecht O (2010). Low light CMOS contact imager with an integrated poly-acrylic emission filter for fluorescence detection. Sensors.

[73] Richard C, Renaudin A, Aimez V, Charette PG (2009). An integrated hybrid interference and absorption filter for fluorescence detection in lab-on-a-chip devices. Lab Chip.

[74] Adams ML, Enzelberger M, Quake S, Scherer A (2003). Microfluidic integration on detector arrays for absorption and fluorescence micro-spectrometers. Sens. Actuators A-Phys..

[75] Hofmann O, Wang X, Cornwell A, Beecher S, Raja A, Bradley DDC, de Mello AJ, de Mello JC (2006). Monolithically integrated dye-doped PDMS long-pass filters for disposable on-chip fluorescence detection. Lab Chip.

[76] Beiderman M, Tam T, Fish A, Jullien GA, Yadid-Pecht O (2008). A low-light CMOS contact imager with an emission filter for biosensing applications. IEEE Trans. Biomed. Circuits Syst..

[77] Pang S, Han C, Lee LM, Yang C (2011). Fluorescence microscopy imaging with a Fresnel zone plate array based optofluidic microscope. Lab Chip.

[78] Lee SA, Ou X, Lee JE, Yang C (2013). Chip-scale fluorescence microscope based on a silo-filter complementary metal-oxide semiconductor image sensor. Opt. Lett..

[79] Hung YJ, Smolyaninov CC Davis, Wu HC (2006). Fluorescence enhancement by surface gratings. Opt. Express.

[80] Gallegos D, Long KD, Yu H, Clark PP, Lin Y, George S, Nath P, Cunningham BT (2013). Label-free biodetection using a smartphone. Lab Chip.

[81] Ricciardi S, Frascella F, Angelini A, Lamberti A, Munzert P, Boarino L, Rizzo R, Tommasi A, Descrovi E (2015). Optofluidic chip for surface wave-based fluorescence sensing. Sens. Actuators B Chem..

[82] Schudel BR, Choi CJ, Cunningham BT, Kenis PJA (2009). Microfluidic chip for combinatorial mixing and screening of assays. Lab Chip.

[83] Danielson TL, Rayson GD, Anderson DM, Estell R, Fredrickson EL, Green BS (2003). Impact of filter paper on fluorescence measurements of buffered saline filtrates. Talanta.

[84] Guo N, Cheung KW, Wong HT, Ho D (2014). CMOS time-resolved, contact, and multispectral fluorescence imaging for DNA molecular diagnostics. Sensors.

[85] Bouccara S, Fragola A, Giovanelli E, Sitbon G, Lequeux N, Pons T, Loriette V (2014). Time-gated cell imaging using long lifetime near-infrared-emitting quantum dots for autofluorescence rejection. J. Biomed. Opt..

[86] Ju Q, Liu Y, Tu D, Zhu H, Li R, Chen X (2011). Lanthanide-doped multicolor GdF3 nanocrystals for time-resolved photoluminescent biodetection. Chem. Eur. J..

[87] Kim H, Petryayeva E, Algar WR (2014). Enhancement of quantum dot Forster resonance energy transfer within paper matrices and application to proteolytic assays. IEEE J. Sel. Top. Quantum Electron..

[88] Paterson AS, Raja B, Mandadi V, Townsend B, Lee M, Buell A, Vu B, Brgoch J, Willson RC (2017). A low-cost smartphone-based platform for highly sensitive point-of-care testing with persistent luminescent phosphors. Lab Chip.

[89] Shah KG, Yager P (2017). Wavelengths and lifetimes of paper autofluorescence: a simple substrate screening process to enhance the sensitivity of fluorescence-based assays in paper. Anal. Chem..

[90] Zhou F, Noor MO, Krull UJ (2014). Luminescence resonance energy transfer-based nucleic acid hybridization assay on cellulose paper with upconverting phosphor as donors. Anal. Chem..

[91] Ispas CR, Crivat G, Andreescu S (2012). Review: recent developments in enzyme-based biosensors for biomedical analysis. Anal. Lett..

[92] He M, Liu Z (2013). Paper-based microfluidic device with upconversion fluorescence assay. Anal. Chem..

[93] Wang L, Yan R, Huo Z, Wang L, Zeng J, Bao J, Wang X, Peng Q, Li Y (2005). Fluorescence resonant energy transfer biosensor based on upconversion-luminescent nanoparticles. Angew. Chem. Int. Ed..

[94] Yu WW, White IM (2013). Inkjet-printed paper-based SERS dipsticks and swabs for trace chemical detection. Analyst.

[95] Qiang J, Uvaraj U, Ulrich K (2014). Paper-based DNA detection using lanthanide-doped LiYF4 upconversion nanocrystals as bioprobe. Small.

[96] Doughan S, Uddayasankar U, Krull UJ (2015). A paper-based resonance energy transfer nucleic acid hybridization assay using upconversion nanoparticles as donors and quantum dots as acceptors. Anal. Chim. Acta.

[97] Golden JP, Ligler FS (2002). A comparison of imaging methods for use in an array biosensor. Biosens. Bioelectron..

[98] B. Jang, P. Cao, A. Chevalier, A. Ellington, A. Hassibi, A CMOS fluorescent-based biosensor microarray. in *2009 IEEE international solid-state circuits conference* (2009), pp. 436–437. 10.1109/isscc.2009.4977495

[99] Giraud G, Schulze H, Li DU, Bachmann TT, Crain J, Tyndall D, Richardson J, Walker R, Stoppa D, Charbon E, Henderson R, Arlt J (2010). Fluorescence lifetime biosensing with DNA microarrays and a CMOS-SPAD imager. Biomed. Opt. Express.

[100] Cetin AE, Coskun AF, Galarreta BC, Huang M, Herman D, Ozcan A, Altug H (2014). Handheld high-throughput plasmonic biosensor using computational on-chip imaging. Light Sci. Appl..

[101] Seo MW, Kagawa K, Yasutomi K, Kawata Y, Teranishi N, Li Z, Halin IA, Kawahito S (2016). A 10 ps time-resolution CMOS image sensor with two-tap true-CDS lock-in pixels for fluorescence lifetime imaging. IEEE J. Solid-State Circuits.

[102] Takehara H, Kazutaka O, Haruta M, Noda T, Sasagawa K, Tokuda T, Ohta J (2017). On-chip cell analysis platform: implementation of contact fluorescence microscopy in microfluidic chips. AIP Adv..

[103] Li W, Knoll T, Sossalla A, Bueth H, Thielecke H (2011). On-chip integrated lensless fluorescence microscopy/spectroscopy module for cell-based sensors. Proc. SPIE.

[104] Kesavan SV, Allier CP, Navarro F, Mittler F, Chalmond B, Dinten JM (2013). Lensless imaging system to quantify cell proliferation. Proc. SPIE.

[105] Zheng G, Lee SA, Antebi Y, Elowitz MB, Yang C (2011). The ePetri dish, an on-chip cell imaging platform based on subpixel perspective sweeping microscopy (SPSM). Proc. Natl. Acad. Sci. USA.

[106] Jain A, Taghavian O, Vallejo D, Dotsey E, Schwartz D, Bell FG, Greef C, Davies DH, Grudzien J, Lee AP, Felgner P, Liang L (2016). Evaluation of quantum dot immunofluorescence and a digital CMOS imaging system as an alternative to conventional organic fluorescence dyes and laser scanning for quantifying protein microarrays. J. Proteom..

